# Lower ventricular and atrial strain in patients who recovered from COVID-19 assessed by cardiovascular magnetic resonance feature tracking

**DOI:** 10.3389/fcvm.2023.1293105

**Published:** 2023-11-13

**Authors:** Mary Luz Mojica-Pisciotti, Roman Panovský, Tomáš Holeček, Lukáš Opatřil, Věra Feitová

**Affiliations:** ^1^ International Clinical Research Center, St. Anne’s University Hospital, Brno, Czech Republic; ^2^ 1st Department of Internal Medicine/Cardioangiology, St. Anne's University Hospital, Brno, Czech Republic; ^3^ Faculty of Medicine, Masaryk University, Brno, Czech Republic; ^4^ Department of Medical Imaging, St. Anne’s University Hospital, Brno, Czech Republic; ^5^ Department of Biomedical Engineering, Brno University of Technology, Brno, Czech Republic

**Keywords:** cardiovascular magnetic resonance, feature tracking, heart deformation, strain analysis, COVID-19

## Abstract

**Introduction:**

One of the most common complications of coronavirus disease 2019 (COVID-19) is myocardial injury, and although its cause is unclear, it can alter the heart's contractility. This study aimed to characterize the ventricular and atrial strain in patients who recovered from COVID-19 using cardiovascular magnetic resonance feature-tracking (CMR-FT).

**Methods:**

In this single-center study, we assessed left ventricle (LV) and right ventricular (RV) global circumferential strain (GCS), global longitudinal strain (GLS), global radial strain (GRS), left atrial (LA) and right atrial (RA) longitudinal strain (LS) parameters by CMR-FT. The student's t-test and Wilcoxon rank-sum test were used to compare the variables.

**Results:**

We compared seventy-two patients who recovered from COVID-19 (49 ± 16 years) to fifty-four controls (49 ± 12 years, *p* = 0.752). The patients received a CMR examination 48 (34 to 165) days after the COVID-19 diagnosis. 28% had LGE. Both groups had normal LV systolic function. Strain parameters were significantly lower in the COVID-19 survivors than in controls.

**Discussion:**

Patients who recovered from COVID-19 exhibited significantly lower strain in the left ventricle (through LVGCS, LVGLS, LVGRS), right ventricle (through RVGLS and RVGRS), left atrium (through LALS), and right atrium (through RALS) than controls.

## 1. Introduction

Coronavirus disease 2019 (COVID-19) is an acute and highly contagious disease caused by the severe acute respiratory syndrome coronavirus 2 (SARS-CoV-2). Although COVID-19 is primarily respiratory, it affects most organs (1) and leads to several cardiac problems (2, 3), including heart failure (4). One of the most common complications is myocardial injury, associated with a worsened prognosis and occurs in 20% to 30% (5, 6).

The cause of cardiac injury is not entirely clear. Current evidence suggests it may be due to excessive inflammatory responses, specifically cytokine release syndrome or “cytokine storm” (7). Other potential mechanisms could include viral myocarditis or pericarditis, stress-induced cardiomyopathy, and microvascular thrombosis (8–11). Regardless of its cause, cardiac injury can alter the heart's contractility, and studying its impact might offer insight into improving post-COVID-19 care.

Cardiovascular magnetic resonance (CMR) has become crucial in diagnosing cardiovascular pathologies and characterizing myocardial tissue and damage (12). In particular, CMR feature tracking (CMR-FT) allows the assessment of the regional deformation of the heart by evaluating the myocardial strain (13, 14). CMR-FT can help to detect subtle systolic or diastolic dysfunction (15), making it an effective tool for assessing cardiovascular health, especially for COVID-19 survivors.

This study aims to characterize the myocardial strain in patients who recovered from COVID-19 with cardiovascular magnetic resonance feature tracking.

## 2. Materials and methods

### 2.1. Study population

A total of 126 subjects, divided into two groups labeled as patients who recovered from COVID-19 (CoV) and a control group (CG), were included in this study. The CoV group comprised 72 patients who had recovered from COVID-19 and underwent a CMR examination after recovery between November 2020 and October 2022. No specific COVID-19 variant was studied. The CG comprised 54 patients of similar sex and age to those in the CoV group, with no history, symptoms, or signs of any pre-existing cardiac disease and negative findings. They were referred to CMR examination for atypical thoracic pain or suspected hypertrophy in those with insufficient echocardiography images. All participants were over 18 years old, had no pre-existence of cardiac disease, chronic inflammatory or autoimmune disease, and no contraindications for CMR examination.

The CoV patients were further divided into two subgroups, group A and group B. Group A consisted of 47 subjects who had recovered from COVID-19 and had a clinical indication for CMR examination. These patients were examined between November 2020 and October 2022 and retrospectively included. Their clinical indication for the CMR examination was not specific to any severity level of COVID-19. Group B consisted of 25 patients prospectively recruited and examined approximately one month after being released from the hospital for being treated for moderate to critical COVID-19 as defined by The National Institute of Health Coronavirus Disease 2019 treatment guidelines (16). These patients exhibited at least one of the following criteria: desaturation with SpO2 ≤ 94%, respiratory frequency ≥30 per minute, lung infiltrates according to x-rays, signs of respiratory failure, and septic shock.

The study is part of a grant project approved by the Ethics Committee of St Anne's University Hospital Brno (Reference Number 6G/2022). It was conducted following the Declaration of Helsinki (2000) of the World Medical Association. All prospectively included participants in the project signed informed consent.

### 2.2. CMR data acquisition

All CMR studies were performed on a 1.5T scanner (Ingenia, Philips Medical Systems) following our standard protocol (17). Cine images were obtained with balanced turbo field echo steady-state free precession (SSFP) sequences (typical parameters: FOV 300 × 300 mm, acquisition voxel size 1.67 × 1.67 × 8.00 mm, reconstruction matrix 256, slice thickness 8 mm, SENSE factor 1.7, 30 to 50 frames per cardiac cycle) in long-axis (two-chamber, four-chamber, three-chamber) and short-axis views. Those were used for functional and strain assessment. Late gadolinium enhancement (LGE) images were acquired approximately 10 min after a contrast bolus injection [0.2 mmol/kg, gadobutrol (Gadovist, Bayer)].

### 2.3. Clinical assessment

An expert radiologist (VF) assessed the left ventricle (LV) and right ventricle (RV) function with the IntelliSpace Portal (ISP) workspace (version 11, Philips Healthcare) according to the established clinical protocols (12). The reported variables were the left ventricle ejection fraction (LVEF), LV end-diastole volume (LVEDV), LV end-systole volume (LVESV), LV stroke volume (LVSV), LV cardiac output (LVCO), LV mass (LVM), right ventricle ejection fraction (RVEF), RV end-diastole volume (RVEDV), RV end-systole volume (RVESV), and RV stroke volume (RVSV). All LV and RV volumes were indexed to the body surface area (BSA), which we indicated by adding the letter I at the end of the abbreviations. Two clinical experts (VF and RP) evaluated LGE and pericardial effusion.

### 2.4. CMR-FT strain assessment

Two experienced readers (MLMP and TH) assessed the LV deformation by 2D strain analysis using the commercial software cvi42 (release 5.13.9, Circle Cardiovascular Imaging). Each reader contoured the endocardial and epicardial LV and RV walls in both the end-diastole (ED) and end-systole (ES) frames in long-axis (two-chamber, four-chamber, three-chamber) and short-axis cine images. They excluded the papillary muscles, epicardial fat, and trabeculae, visually verified each contour, and adjusted if necessary. Only images from the short-axis stack free from the left ventricular outflow tract were considered in the analysis. The software automatically propagated the contours and determined the global longitudinal strain (GLS), global circumferential strain (GCS), and global radial strain from both SAX (GRS_SAX_) and LAX images (GRS_LAX_). GRS was the average of GRS_SAX_ and GRS_LAX_.

Likewise, the readers analyzed the left atrium (LA) and right atrium (RA) with the same software. They traced the LA and RA contours in the ED and ES frames in two- and four-chamber long-axis images. The software determined the atrial strain by averaging the measurements from these contours. The results included the following LA and RA parameters: minimum volume (LAVmin, RAVmin), maximum volume (LAVmax, RAVmax), ejection fraction (LAEF, RAEF), and longitudinal strain (LALS, RALS). LA and RA volumes were indexed to the BSA.

### 2.5. Statistical analysis

Descriptive statistics are reported as the mean (standard deviation, SD) or median (interquartile range, IQR) for normally and non-normally distributed continuous variables and as numbers (percentages) for categorical ones. The normality of the data was checked by the Shapiro-Wilk test and visual inspection of the histograms. Proportions of categorical variables were analyzed using the Chi-square test of independence. The student's t-test and Wilcoxon rank-sum test were used to compare normally and non-normally distributed variables. The adjusted *P*-value was obtained using a false discovery rate correction to account for multiple comparisons. A *P*-value < 0.05 was considered statistically significant. The intraobserver and interobserver agreement was assessed with the intraclass correlation coefficient (ICC). The ICC (two-way mixed-effects model) was determined from twenty randomly selected cases analyzed by two readers (MLMP, TH), one of whom repeated them one month apart. The repeatability was classified as poor (<0.5), fair (0.50 to 0.75), good (0.75 to 0.90), and excellent (0.90 to 1) (18). All statistical analyses were performed with R-4.2.2 and RStudio IDE (2022.12.0 + 353, RStudio, PBC).

## 3. Results

### 3.1. Study group

**Figure 1:**
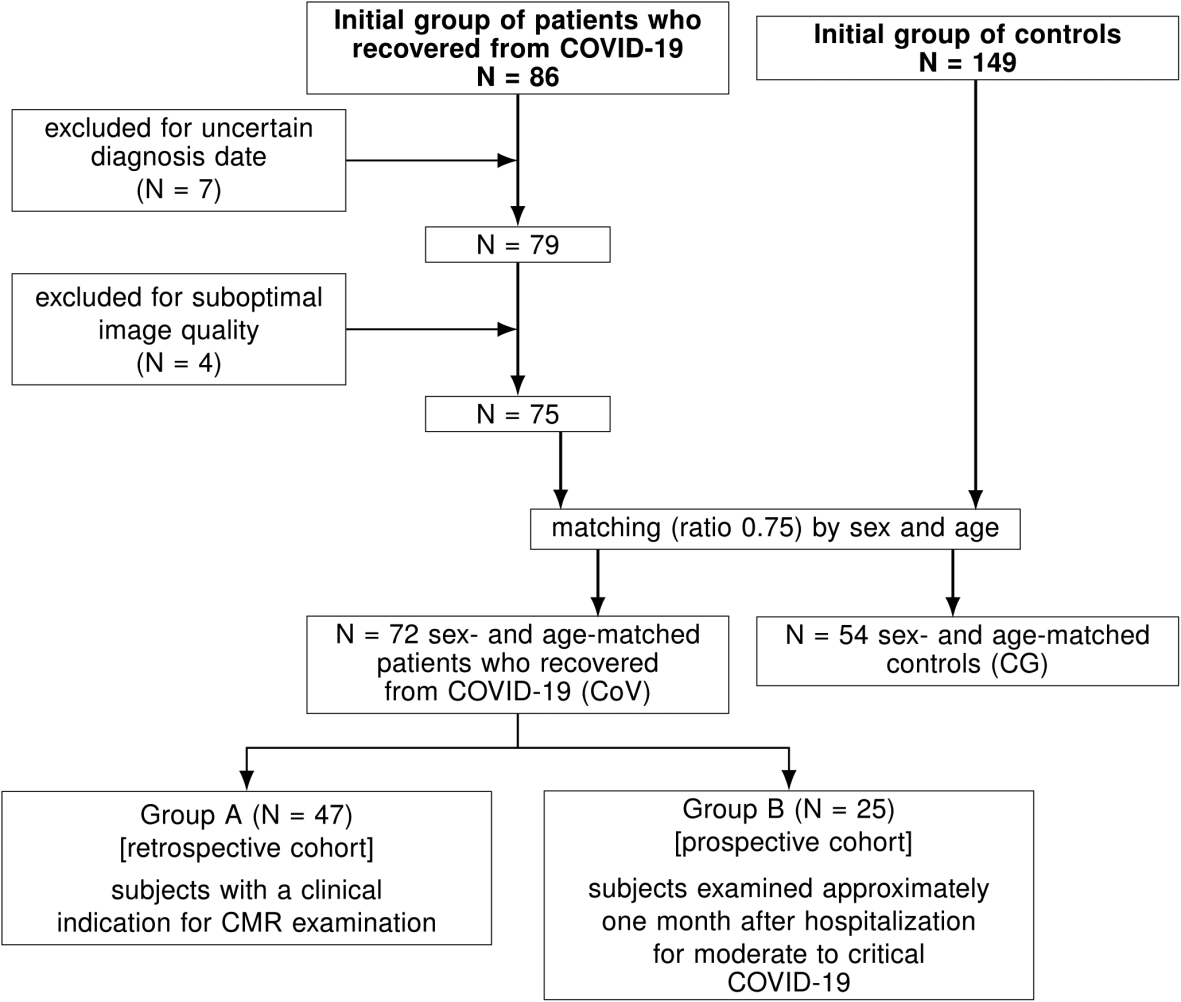
Study flowchart. CMR, cardiovascular magnetic resonance.

The study flowchart is shown in Figure 1. General characteristics were similar in both groups (see Table 1). The median time between the COVID-19 diagnosis and the CMR examination was 48 (34 to 165) days. Although both groups had normal LVEF, significantly lower LVEF, RVEF, and LAEF were found in patients who recovered from COVID-19 compared to CG. On the contrary, significantly higher LAVImin, RAVImin, and LVESVI were found in the CoV group than in CG. Additionally, 28% of CoV (*n* = 20) had LGE, and 3% (*n* = 2) had pericardial effusion (≥10 mm). The LGE patterns were non-ischemic, in most cases, subepicardial (*n* = 12), mid-myocardial (*n* = 4), transmural (*n* = 2), and subendocardial (*n* = 2) mainly found in the basal segment of anterolateral or inferolateral walls.

**Table 1:** Demographics and clinical parameters in patients who recovered from COVID-19 (CoV) and a control group (CG).

Variable	CG, *N* = 54	CoV, *N* = 72	*P*-value
Age (y)	49 (12)	49 (16)	0.752
Sex (female/male)	26 / 28	33 / 39	0.938
BMI (kg/m^2^)	26.7 (23.6, 28.9)	27.3 (24.2, 30.5)	0.171
BSA (m^2^)	1.98 (0.22)	1.98 (0.20)	0.998
HR (bpm)	64 (57, 69)	72 (63, 78)	**0.005**
LGE+ (n, %)		20 (28%)	
Pericardial effusion (≥10 mm) (*n*, %)		2 (3%)	
Left ventricle			
LVEF (%)	66 (63, 73)	63 (58, 70)	**<0.001**
LVEDVI (ml/m^2^)	60.4 (55.3, 66.0)	64.8 (55.4, 74.2)	0.080
LVESVI (ml/m^2^)	19.7 (14.9, 24.2)	23.3 (15.8, 31.5)	**0.002**
LVSVI (ml/m^2^)	41.5 (6.5)	39.5 (8.5)	0.139
CI (l/min/m^2^)	2,618.4 (2,318.9, 2,877.9)	2,878.7 (2,376.8, 3,170.8)	0.423
LVMI (g/m^2^)	44.7 (38.0, 54.0)	49.3 (41.2, 60.5)	0.135
Right ventricle			
RVEF (%)	64 (8)	60 (10)	**0.034**
RVEDVI (ml/m^2^)	66.2 (13.7)	67.0 (17.2)	0.751
RVESVI (ml/m^2^)	22.8 (18.0, 30.1)	26.5 (17.6, 36.9)	0.123
RVSVI (ml/m^2^)	41.5 (7.1)	39.3 (8.1)	0.120
Left atrium			
LAVImin (ml/m^2^)	12.2 (9.6, 16.0)	12.6 (10.8, 19.2)	**0.008**
LAVImax (ml/m^2^)	30.9 (25.0, 36.4)	31.7 (24.3, 38.2)	0.469
LAEF (%)	62 (56, 66)	57 (48, 63)	**0.001**
Right atrium			
RAVImin (ml/m^2^)	18.1 (13.4, 23.5)	16.4 (11.1, 22.5)	0.700
RAVImax (ml/m^2^)	39.9 (11.0)	35.2 (12.3)	**0.025**
RAEF (%)	54 (46, 58)	53 (42, 60)	0.076

Variables are expressed as numbers/total (percentages), mean (standard deviation), or median (interquartile range) for categorical, normally distributed, and non-normally distributed continuous variables.

BMI, body mass index; BSA, body surface area; CG, control group; CI, cardiac index; CoV, patients who recovered from COVID-19; EF, ejection fraction; EDV, end-diastole volume; ESV, end-systole volume; HR, heart rate; I, indexed; LA, left atrium; LGE, late gadolinium enhancement; LV, left ventricle; LVCO, left ventricular cardiac output; LVM, left ventricular mass; max, maximum; min, minimum; n, number of subjects; N, total number of subjects; RA, right atrium; RV, right ventricle; SV, stroke volume.

*p*-values <0.05 are presented in bold.

In the patients treated for moderate to critical COVID-19 (Group B), we found anemia, renal insufficiency, and appetite loss in 4% (1/25), diabetes mellitus in 8% (2/25), hypertension in 20% (5/25), and dyslipidemia in 12% (3/25). Also, cardiac biomarkers during hospitalization for this group were measured: NTproBNP 124 (55, 341) ng/L (20 patients), troponin 7 (5, 10) ng/L (17 patients), highest CPR 120 (57, 185) mg/L, highest leucocytes 9.5 (6.6, 11.2) cells/*µ*l, glomerular filtration 1.17 ± 0.47 ml/s/1.73 m^2^, and creatinin 87 (79, 120) *µ*mol/L.

### 3.2. Strain parameters

**Figure 2:**
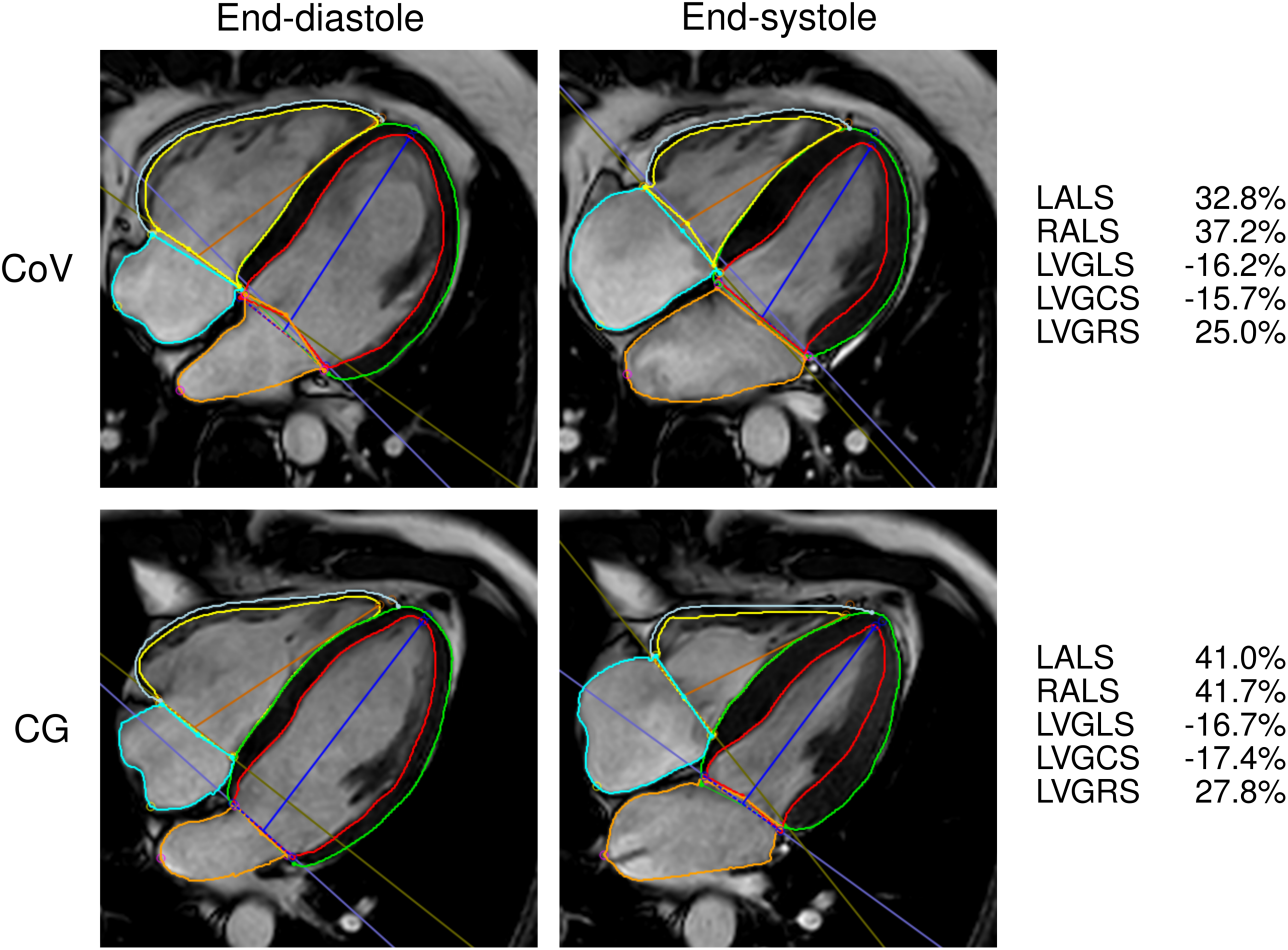
Contouring for the ventricular and atrial strain calculation in one patient who recovered from COVID-19 (CoV) and one control (CG). GCS, global circumferential strain; GLS, global longitudinal strain; GRS, global radial strain; LA, left atrium; LS, longitudinal strain; LV, left ventricle; RA, right atrium; RV, right ventricle.

Ventricular and atrial strain parameters were significantly lower in the CoV group than in CG, except for the RVGCS and RVGRS_SAX_ (see Table 2). A comparison of the strain assessment is shown in Figure 2.

**Table 2:** Left ventricular, right ventricular, left atrial and right atrial strain in patients who recovered from COVID-19 (CoV) and a control group (CG).

Variable	CG, *N* = 54	CoV, *N* = 72	*P*-value
Left ventricle			
LVGCS (%)	−17.9 (−19.6, −16.9)	−17.0 (−18.5, −14.9)	**<0.001**
LVGLS (%)	−17.8 (−19.3, −16.8)	−17.4 (−18.6, −15.4)	**0.002**
LVGRS_LAX_ (%)	32.1 (29.7, 36.1)	29.4 (25.3, 33.1)	**<0.001**
LVGRS_SAX_ (%)	29.0 (26.9, 33.6)	27.1 (22.5, 30.8)	**<0.001**
LVGRS (%)	31.2 (28.0, 33.9)	28.5 (24.1, 31.8)	**<0.001**
Right ventricle			
RVGCS (%)	−14.8 (4.2)	−13.7 (4.5)	0.157
RVGLS (%)	−25.9 (−28.3, −23.0)	−24.4 (−26.5, −22.0)	**0.048**
RVGRS_LAX_ (%)	59.5 (47.3, 74.2)	53.5 (46.8, 61.2)	**0.027**
RVGRS_SAX_ (%)	23.7 (19.0, 32.6)	21.9 (17.0, 26.3)	0.124
RVGRS (%)	42.6 (35.8, 48.60)	38.3 (32.7, 44.2)	**0.017**
Left atrium			
LALS (%)	36.6 (29.5, 47.9)	28.8 (19.5, 41.0)	**0.003**
Right atrium			
RALS (%)	43.9 (14.4)	37.9 (17.8)	**0.037**

Variables are expressed as mean (standard deviation) or median (interquartile range) for normally distributed and non-normally distributed continuous variables.

CG, control group; CoV, patients who recovered from COVID-19; GCS, global circumferential strain; GLS, global longitudinal strain; GRS, global radial strain; LA, left atrium; LAX, long axis; LS, longitudinal strain; LV, left ventricle; RA, right atrium; RV, right ventricle; SAX, short axis.

*p*-values <0.05 are presented in bold.

### 3.3. Comparison between two groups of patients who recovered from COVID-19

General parameters were similar between the two subgroups of patients who recovered from COVID-19 (see Table 3). We compared the groups considering three age groups (<50, 50 to 70, and >70 years) and found no significant differences in the cardiac strain (see Supplementary Table S1).

**Table 3:** Demographics and clinical parameters in patients who recovered from COVID-19: group A (with a clinical CMR indication after recovery) and group B (treated for moderate to critical COVID-19).

Variable	Overall, *N* = 72	Group A, *N* = 47	Group B, *N* = 25	*P*-value
Age (y)	49 (16)	47 (16)	55 (15)	**0.039**
Sex (female/male)	33 / 39	18 / 29	15 / 10	0.131
BMI (kg/m^2^)	27.3 (24.2, 30.5)	27.1 (24.1, 30.4)	29.0 (24.8, 30.6)	0.567
BSA (m^2^)	1.98 (0.20)	1.99 (0.22)	1.96 (0.17)	0.538
HR (bpm)	72 (14)	72 (14)	70 (12)	0.526
LGE+ (*n*, %)	20 (28%)	17 (36%)	3 (12%)	**0.029**
Time between diagnosis and CMR (days)	48 (34, 165)	76 (40, 222)	37 (34, 46)	**<0.001**
Left ventricle				
LVEF (%)	63 (58, 70)	62 (55, 68)	66 (61, 70)	**0.011**
LVEDVI (ml/m^2^)	65.8 (17.1)	69.4 (16.9)	59.1 (15.8)	**0.012**
LVESVI (ml/m^2^)	23.3 (15.8, 31.5)	26.0 (18.8, 32.6)	18.7 (13.7, 25.7)	**0.004**
LVSVI (ml/m^2^)	39.5 (8.5)	39.9 (8.1)	38.7 (9.3)	0.605
CI (l/min/m^2^)	2,798.2 (552.2)	2,867.4 (565.5)	2,668.0 (512.1)	0.135
LVMI (g/m^2^)	51.3 (14.3)	52.6 (15.2)	48.9 (12.1)	0.267
Right ventricle				
RVEF (%)	60 (10)	59 (10)	63 (9)	0.093
RVEDVI (ml/m^2^)	67.0 (17.2)	69.6 (17.0)	62.3 (16.9)	0.089
RVESVI (ml/m^2^)	26.5 (17.6, 36.9)	28.2 (17.7, 37.8)	23.8 (15.7, 31.6)	0.055
RVSVI (ml/m^2^)	39.3 (8.1)	39.9 (7.6)	38.2 (9.0)	0.423
Left atrium				
LAVImin (ml/m^2^)	12.6 (10.8, 19.2)	12.6 (10.9, 20.0)	12.3 (7.8, 17.5)	**0.048**
LAVImax (ml/m^2^)	31.7 (24.3, 38.2)	31.2 (25.5, 38.3)	32.2 (20.3, 37.9)	0.270
LAEF (%)	57 (48, 63)	56 (42, 61)	59 (53, 63)	**0.017**
Right atrium				
RAVImin (ml/m^2^)	16.4 (11.1, 22.5)	17.8 (11.5, 23.3)	13.4 (10.4, 20.3)	0.101
RAVImax (ml/m^2^)	35.2 (12.3)	35.8 (12.8)	34.0 (11.4)	0.542
RAEF (%)	53 (42, 60)	50 (36, 60)	59 (49, 63)	**0.006**

Variables are expressed as numbers/total (percentages), mean (standard deviation), or median (interquartile range) for categorical, normally distributed, and non-normally distributed continuous variables.

BMI, body mass index; BSA, body surface area; CI, cardiac index; EF, ejection fraction; EDV, end-diastole volume; ESV, end-systole volume; HR, heart rate; I, indexed; LA, left atrium; LGE, late gadolinium enhancement; LV, left ventricle; LVCO, left ventricular cardiac output; LVM, left ventricular mass; max, maximum; min, minimum; n, number of subjects; N, total number of subjects; RA, right atrium; RV, right ventricle; SV, stroke volume.

*p*-values <0.05 are presented in bold.

### 3.4. Reproducibility

The intraobserver and interobserver reproducibility was good or excellent for most strain parameters and fair for the interobserver RVGRS_SAX_ (see Table 4).

**Table 4:** Intraobserver and interobserver reproducibility (ICC, two-way mixed-effects model) of left ventricular, right ventricular, left atrial, and right atrial strain.

Variable	Intraobserver	Interobserver
LVGCS (%)	0.995 (0.986–0.998)	0.998 (0.994–0.999)
LVGLS (%)	0.996 (0.989–0.998)	0.996 (0.986–0.999)
LVGRS_LAX_ (%)	0.995 (0.989–0.998)	0.994 (0.980–0.998)
LVGRS_SAX_ (%)	0.993 (0.982–0.997)	0.998 (0.994–0.999)
RVGCS (%)	0.837 (0.634–0.932)	0.808 (0.461–0.941)
RVGLS (%)	0.959 (0.900–0.984)	0.940 (0.807–0.982)
RVGRS_LAX_ (%)	0.948 (0.874–0.979)	0.925 (0.762–0.978)
RVGRS_SAX_ (%)	0.825 (0.609–0.927)	0.675 (0.195–0.894)
LALS (%)	0.972 (0.930–0.989)	0.960 (0.867–0.988)
RALS (%)	0.831 (0.622–0.930)	0.973 (0.909–0.992)

Values are expressed as ICC (95% CI).

CI, confidence interval; GCS, global circumferential strain; GLS, global longitudinal strain; GRS, global radial strain; ICC, intraclass correlation coefficient; LA, left atrium; LAX, long axis; LS, longitudinal strain; LV, left ventricle; RA, right atrium; RV, right ventricle; SAX, short axis.

## 4. Discussion

We assessed CMR-derived ventricular and atrial strain parameters in patients who recovered from COVID-19 and found significantly lower cardiac strain values compared to a control group. As far as we know, this study is the first to report lower atrial longitudinal strain values in this population.

Some studies reported lower values of GLS and GCS in patients who recovered from COVID-19 than in healthy controls (19–23). However, this difference was only significant in a handful (21–23). Both parameters were lower in patients who recovered from the delta variant of COVID-19 (23). Lower GLS was found in patients who recovered from severe or moderate cases (21) and those with LGE (22). No significant alterations in GRS values have been reported so far in these patients (21–24). In our study, we included patients regardless of their COVID-19 variant.

The improvement of cardiac function in patients who recovered from COVID-19 seems time-dependent. A study reported that 30 days after the initial COVID-19 diagnosis, the GLS could detect subclinical LV dysfunction in patients with low cardiac risk (25). Another one, in patients during the acute phase of COVID-19 infection, showed abnormal myocardial mechanics within 3 to 8 days after the diagnosis, revealing significantly lower peak GLS than controls and a low rate of positive LGE (4%) (26).

In our study, the group of patients examined around 30 days after recovering from COVID-19 had similar cardiac strain to those examined later. In this group, we did not study the severity of the COVID-19 symptoms during the active phase of the disease or its connection to cardiac outcomes. It has been shown that mild cases of COVID-19 experience fewer complications and take less time to recover than more severe cases (27–29). There is also compelling evidence of cardiovascular sequelae among survivors beyond the first month of the illness (30).

So far, only one published study reported similar CMR-derived LA and RA strains between patients with persistent symptoms (such as arrhythmia, fatigue, weakness, and lack of taste or smell) examined three months after a positive test for COVID-19 infection and healthy volunteers (20). Contrary to our study, our patients had no similar persistent disease symptoms and were examined earlier than three months after the diagnosis. We also identified lower global LV strains in our cohort, which may account for these two studies' differences. The severity of the COVID-19 symptoms in the participants of both studies was not compared, which could also influence cardiac function recovery (21).

We found LGE in 28% of the patients who recovered from COVID-19, which agrees with other studies (22, 24, 31, 32). However, we did not observe as many mid-myocardial or patchy patterns as other authors (22), but instead, mainly subepicardial involvement.

Cardiac strain is assessed through Speckle-Tracking echocardiography (STE) or CMR-FT (14, 33). Although STE is more available and portable than CMR, it is frequently affected by motion artifacts and has a lower spatial resolution than CMR-FT (14). Nonetheless, there is evidence of a good agreement between the assessment of GLS and GCS using STE and CMR-FT (14). On the other hand, there has been a growing interest in assessing atrial function using STE. Although such a possibility dramatically depends on high-quality images and is challenging because of the narrow walls of the atria, some studies support its feasibility and comparability with CMR-FT (34–36).

Regarding patients who recovered from COVID-19, echocardiography-based studies reported lower GLS in those examined within two months of the diagnosis (25, 37) and a slight improvement after three months of the hospital discharge (38). The GLS reaches similar levels to those in controls around six months after the diagnosis (39–41). One study reported similar LA and RA peak systolic strain values between COVID-19 survivors and healthy controls (39).

## 5. Limitations

This single-center study has some limitations. Firstly, our study population was relatively small, primarily due to the unprecedented demand for healthcare during the first waves of the COVID-19 pandemic. Such conditions translated into prioritizing other imaging modalities over CMR to treat affected individuals. The hospital strain posed a lower availability of CMR equipment. Secondly, most sufferers in our cohort did not exhibit severe enough symptoms, had previous pathologies, or had low compliance to participate in research. Therefore, our results may not apply to the complete range of patients who recovered from COVID-19. Thirdly, we lack the clinical biomarkers assessment for patients with a clinical CMR indication after recovery (Group A), as they were retrospectively recruited. Also, we could not ascertain the specific COVID-19 variant for each patient. Fourthly, we did not study the severity of the COVID-19 symptoms during the active phase of the disease, its connection to cardiac outcomes, or the influence of other cardiovascular conditions, such as heart failure (4). Also, we had no baseline CMR examination for the participants, meaning we cannot directly link the decreased strain values to the COVID-19 infection. Finally, we did not perform phasic atrial strain assessment as the cvi42 software is not optimized for this task.

## 6. Conclusions

We conclude that patients who recovered from COVID-19 exhibited significantly lower strain in the left ventricle (through GCS, GLS, GRS), right ventricle (through RVGLS and RVGRS), left atrium (through LALS), and right atrium (through RALS) than controls.

## Data Availability

The raw data supporting the conclusions of this article will be made available by the authors, without undue reservation.

## References

[1] Raman B Cassar MP Tunnicliffe EM Filippini N Griffanti L Alfaro-Almagro F Medium-term effects of SARS-CoV-2 infection on multiple vital organs, exercise capacity, cognition, quality of life and mental health, post-hospital discharge. EClinicalMedicine. (2021) 31:100683. 10.1016/j.eclinm.2020.100683 33490928 PMC7808914

[2] Babapoor-Farrokhran S Gill D Walker J Rasekhi RT Bozorgnia B Amanullah A. Myocardial injury and COVID-19: possible mechanisms. Life Sci. (2020) 253:117723. 10.1016/j.lfs.2020.117723 32360126 PMC7194533

[3] Chang WT Toh HS Liao CT Yu WL. Cardiac involvement of COVID-19: a comprehensive review. Am J Med Sci. (2021) 361(1):14–22. 10.1016/j.amjms.2020.10.002 33187633 PMC7536131

[4] Yonas E Alwi I Pranata R Huang I Lim MA Gutierrez EJ Effect of heart failure on the outcome of COVID-19 — a meta analysis and systematic review. Am J Emerg Med. (2021) 46:204–11. 10.1016/j.ajem.2020.07.009 33071085 PMC7347316

[5] Yang X Yu Y Xu J Shu H Ja X Liu H Clinical course and outcomes of critically ill patients with SARS-CoV-2 pneumonia in Wuhan, China: a single-centered, retrospective, observational study. Lancet Respir Med. (2020) 8(5):475–81. 10.1016/S2213-2600(20)30079-5 32105632 PMC7102538

[6] Shi S Qin M Cai Y Liu T Shen B Yang F Characteristics and clinical significance of myocardial injury in patients with severe coronavirus disease 2019. Eur Heart J. (2020) 41(22):2070–9. 10.1093/eurheartj/ehaa408 32391877 PMC7239100

[7] Wang Y Zheng J Islam MS Yang Y Hu Y Chen X. The role of CD4(+)FoxP3(+) regulatory T cells in the immunopathogenesis of COVID-19: implications for treatment. Int J Biol Sci. (2021) 17(6):1507–20. 10.7150/ijbs.59534 33907514 PMC8071774

[8] Hendren NS Drazner MH Bozkurt B Cooper LT. Description and proposed management of the acute COVID-19 cardiovascular syndrome. Circulation. (2020) 141(23):1903–14. 10.1161/CIRCULATIONAHA.120.047349 32297796 PMC7314493

[9] Tavazzi G Pellegrini C Maurelli M Belliato M Sciutti F Bottazzi A Myocardial localization of coronavirus in COVID-19 cardiogenic shock. Eur J Heart Fail. (2020) 22(5):911–5. 10.1002/ejhf.1828 32275347 PMC7262276

[10] Bavishi C Bonow RO Trivedi V Abbott JD Messerli FH Bhatt DL. Special article—acute myocardial injury in patients hospitalized with COVID-19 infection: a review. Prog Cardiovasc Dis. (2020) 63(5):682–9. 10.1016/j.pcad.2020.05.013 32512122 PMC7274977

[11] Kotecha T Knight DS Razvi Y Kumar K Vimalesvaran K Thornton G Patterns of myocardial injury in recovered troponin-positive COVID-19 patients assessed by cardiovascular magnetic resonance. Eur Heart J. (2021) 42(19):1866–78. 10.1093/eurheartj/ehab075 33596594 PMC7928984

[12] Schulz-Menger J Bluemke DA Bremerich J Flamm SD Fogel MA Friedrich MG Standardized image interpretation and post-processing in cardiovascular magnetic resonance—2020 update. J Cardiovasc Magn Reson. (2020) 22(1):19. 10.1186/s12968-020-00610-6 32160925 PMC7066763

[13] Schuster A Hor KN Kowallick JT Beerbaum P Kutty S. Cardiovascular magnetic resonance myocardial feature tracking: concepts and clinical applications. Circ Cardiovasc Imaging. (2016) 9(4):e004077. 10.1161/CIRCIMAGING.115.004077 27009468

[14] Pedrizzetti G Claus P Kilner PJ Nagel E. Principles of cardiovascular magnetic resonance feature tracking and echocardiographic speckle tracking for informed clinical use. J Cardiovasc Magn Reson. (2016) 18(1):51. 10.1186/s12968-016-0269-7 27561421 PMC5000424

[15] Kermer J Traber J Utz W Hennig P Menza M Jung B Assessment of diastolic dysfunction: comparison of different cardiovascular magnetic resonance techniques. ESC Heart Failure. (2020) 7(5):2637–49. 10.1002/ehf2.12846 32686332 PMC7524101

[16] The National Institute of Health Coronavirus Disease 2019 (COVID-19) treatment guidelines. Available at: www.covid19treatmentguidelines.nih.gov 34003615

[17] Panovský R Pešl M Máchal J Holeček T Feitová V Juříková L Quantitative assessment of left ventricular longitudinal function and myocardial deformation in Duchenne muscular dystrophy patients. Orphanet J Rare Dis. (2021) 16(1):57. 10.1186/s13023-021-01704-9 33516230 PMC7847593

[18] Koo TK Li MY. A guideline of selecting and reporting intraclass correlation coefficients for reliability research. J Chiropr Med. (2016) 15(2):155–63. 10.1016/j.jcm.2016.02.012 27330520 PMC4913118

[19] Myhre PL Heck SL Skranes JB Prebensen C Jonassen CM Berge T Cardiac pathology 6 months after hospitalization for COVID-19 and association with the acute disease severity. Am Heart J. (2021) 242:61–70. 10.1016/j.ahj.2021.08.001 34400140 PMC8363180

[20] Tanacli R Doeblin P Götze C Zieschang V Faragli A Stehning C COVID-19 vs. classical myocarditis associated myocardial injury evaluated by cardiac magnetic resonance and endomyocardial biopsy. Front Cardiovasc Med. (2021) 8:737257. 10.3389/fcvm.2021.737257 35004872 PMC8739473

[21] Li X Wang H Zhao R Wang T Zhu Y Qian Y Elevated extracellular volume fraction and reduced global longitudinal strains in participants recovered from COVID-19 without clinical cardiac findings. Radiology. (2021) 299(2):E230–e40. 10.1148/radiol.2021203998 33434112 PMC7808090

[22] Wang H Li R Zhou Z Jiang H Yan Z Tao X Cardiac involvement in COVID-19 patients: mid-term follow up by cardiovascular magnetic resonance. J Cardiovasc Magn Reson. (2021) 23(1):14. 10.1186/s12968-021-00710-x 33627143 PMC7904320

[23] Zhang L Wei X Wang H Jiang R Tan Z Ouyang J Cardiac involvement in patients recovering from delta variant of COVID-19: a prospective multi-parametric MRI study. ESC Heart Failure. (2022) 9(4):2576–84. 10.1002/ehf2.13971 35560820 PMC9288765

[24] Urmeneta Ulloa J Martínez de Vega V Salvador Montañés O Álvarez Vázquez A Sánchez-Enrique C Hernández Jiménez S Cardiac magnetic resonance in recovering COVID-19 patients. Feature tracking and mapping analysis to detect persistent myocardial involvement. Int J Cardiol Heart Vasc. (2021) 36:100854. 10.1016/j.ijcha.2021.100854 34368419 PMC8328575

[25] Turan T Özderya A Şahin S Konuş AH Kul S Akyüz AR Left ventricular global longitudinal strain in low cardiac risk outpatients who recently recovered from coronavirus disease 2019. Int J Cardiovasc Imaging. (2021) 37(10):2979–89. 10.1007/s10554-021-02376-z 34387799 PMC8360821

[26] Chen B-H Shi N-N Wu C-W An D-A Shi Y-X Wesemann LD Early cardiac involvement in patients with acute COVID-19 infection identified by multiparametric cardiovascular magnetic resonance imaging. Eur Heart J Cardiovasc Imaging. (2021) 22(8):844–51. 10.1093/ehjci/jeab042 33686389 PMC7989521

[27] Ayoubkhani D Khunti K Nafilyan V Maddox T Humberstone B Diamond I Post-COVID syndrome in individuals admitted to hospital with COVID-19: retrospective cohort study. BMJ (Clin Res). (2021) 372:n693. 10.1136/bmj.n693 PMC8010267 33789877

[28] Xie Y Xu E Bowe B Al-Aly Z. Long-term cardiovascular outcomes of COVID-19. Nat Med. (2022) 28(3):583–90. 10.1038/s41591-022-01689-3 35132265 PMC8938267

[29] Puntmann VO Martin S Shchendrygina A Hoffmann J Ka MM Giokoglu E Long-term cardiac pathology in individuals with mild initial COVID-19 illness. Nat Med. (2022) 28(10):2117–23. 10.1038/s41591-022-02000-0 36064600 PMC9556300

[30] Al-Aly Z Xie Y Bowe B. High-dimensional characterization of post-acute sequelae of COVID-19. Nature. (2021) 594(7862):259–64. 10.1038/s41586-021-03553-9 33887749

[31] Puntmann VO Carerj ML Wieters I Fahim M Arendt C Hoffmann J Outcomes of cardiovascular magnetic resonance imaging in patients recently recovered from coronavirus disease 2019 (COVID-19). JAMA Cardiology. (2020) 5(11):1265–73. 10.1001/jamacardio.2020.3557 32730619 PMC7385689

[32] Huang L Zhao P Tang D Zhu T Han R Zhan C Cardiac involvement in patients recovered from COVID-2019 identified using magnetic resonance imaging. JACC Cardiovasc Imaging. (2020) 13(11):2330–9. 10.1016/j.jcmg.2020.05.004 32763118 PMC7214335

[33] Smiseth OA Torp H Opdahl A Haugaa KH Urheim S. Myocardial strain imaging: how useful is it in clinical decision making? Eur Heart J. (2016) 37(15):1196–207. 10.1093/eurheartj/ehv529 26508168 PMC4830908

[34] Kowallick JT Lotz J Hasenfuß G Schuster A. Left atrial physiology and pathophysiology: role of deformation imaging. World J Cardiol. (2015) 7(6):299–305. 10.4330/wjc.v7.i6.299 26131333 PMC4478563

[35] Truong VT Palmer C Young M Wolking S Ngo TNM Sheets B Right atrial deformation using cardiovascular magnetic resonance myocardial feature tracking compared with two-dimensional speckle tracking echocardiography in healthy volunteers. Sci Rep. (2020) 10(1):5237. 10.1038/s41598-020-62105-9 32251322 PMC7089993

[36] Badano LP Kolias TJ Muraru D Abraham TP Aurigemma G Edvardsen T Standardization of left atrial, right ventricular, and right atrial deformation imaging using two-dimensional speckle tracking echocardiography: a consensus document of the EACVI/ASE/industry task force to standardize deformation imaging. Eur Heart J Cardiovasc Imaging. (2018) 19(6):591–600. 10.1093/ehjci/jey042 29596561

[37] Lassen MCH Skaarup KG Lind JN Alhakak AS Sengeløv M Nielsen AB Recovery of cardiac function following COVID-19—eCHOVID-19: a prospective longitudinal cohort study. Eur J Heart Fail. (2021) 23(11):1903–12. 10.1002/ejhf.2347 34514713 PMC8652600

[38] Ingul CB Grimsmo J Mecinaj A Trebinjac D Berger Nossen M Andrup S Cardiac dysfunction and arrhythmias 3 months after hospitalization for COVID-19. J Am Heart Assoc. (2022) 11(3):e023473-e. 10.1161/JAHA.121.023473 35048715 PMC9238505

[39] Akbulut M Tan S Gerede Uludağ DM Kozluca V Dinçer İ. Evaluation of cardiac function in uncomplicated COVID-19 survivors by 2-dimensional speckle tracking imaging. Anatol J Cardiol. (2022) 26(11):841–8. 10.5152/AnatolJCardiol.2022.1360 35949116 PMC9682558

[40] Øvrebotten T Myhre P Grimsmo J Mecinaj A Trebinjac D Nossen MB Changes in cardiac structure and function from 3 to 12 months after hospitalization for COVID-19. Clin Cardiol. (2022) 45(10):1044–52. 10.1002/clc.23891 35920837 PMC9538691

[41] Gao Y-P Zhou W Huang P-N Liu H-Y Bi X-J Zhu Y Normalized cardiac structure and function in COVID-19 survivors late after recovery. Front Cardiovasc Med. (2021) 8:756790. 10.3389/fcvm.2021.756790 34912863 PMC8666962

