# Supplementary material for: Lower ventricular and atrial strain in patients who recovered from COVID-19 assessed by cardiovascular magnetic resonance feature tracking
Source: Front Cardiovasc Med. 2023 Nov 13;10:1293105. doi: 10.3389/fcvm.2023.1293105 (PMC10679333; doi:10.3389/fcvm.2023.1293105)
Supplement: Supplementary file 1 [file Table1.docx]

Supplementary Material

**Supplementary Table 1.** Cardiac strain in patients who recovered from COVID-19 (CoV): Group A (patients with a clinical CMR indication after recovery) and Group B (patients treated for moderate to critical COVID-19) by age group.

|  | **Age < 50, N = 37** | | | **Age 50-70, N = 27** | | | **Age > 70, N = 8** | | |
| --- | --- | --- | --- | --- | --- | --- | --- | --- | --- |
| **Variable** | **Group A, N = 28** | **Group B, N = 9** | **Adj. P-value** | **Group A, N = 14** | **Group B, N = 13** | **Adj. P-value** | **Group A, N = 5** | **Group B, N = 3** | **Adj. P-value** |
| Age (years) | 36 (9) | 37 (6) | 0.882 | 58.8 (5.5) | 62.6 (5.5) | 0.572 | 74.81 (4.32) | 72.17 (2.19) | 0.591 |
| Sex (female/male) | 10 / 18 | 3 / 6 | >0.999 | 7 / 7 | 9 / 4 | 0.695 | 1 / 4 | 3 / 0 | 0.591 |
| Time between diagnosis and CMR (days) | 106 (39, 213) | 44 (41, 71) | **0.045** | 113 (51, 310) | 36 (33, 45) | 0.205 | 40 (31, 41) | 34 (32, 34) | 0.591 |
| **Left ventricle** |  |  |  |  |  |  |  |  |  |
| LVGCS (%) | -16.4 (-17.6, -14.1) | -16.8 (-18.3, -16.1) | 0.580 | -16.1 (3.7) | -17.6 (2.1) | 0.572 | -15.8 (2.5) | -18.6 (1.4) | 0.591 |
| LVGLS (%) | -17.0 (-18.3, -14.2) | -17.3 (-18.0, -15.9) | 0.806 | -16.6 (3.6) | -17.8 (1.7) | 0.572 | -17.4 (-17.9, -16.2) | -18.0 (-18.8, -17.8) | 0.591 |
| LVGRS_SAX_ (%) | 25.9 (20.3, 29.0) | 27.1 (25.1, 30.0) | 0.806 | 26.0 (8.5) | 28.7 (5.0) | 0.572 | 25.1 (5.7) | 31.0 (3.5) | 0.591 |
| LVGRS_LAX_ (%) | 26.8 (9.5) | 28.7 (4.9) | 0.882 | 28.8 (9.2) | 31.6 (4.7) | 0.572 | 27.8 (7.3) | 31.9 (4.2) | 0.591 |
| LVGRS (%) | 27 (22, 30) | 30 (25, 31) | 0.806 | 27 (9) | 30 (4) | 0.572 | 26.5 (5.9) | 31.4 (3.1) | 0.591 |
| **Right ventricle** |  |  |  |  |  |  |  |  |  |
| RVGCS (%) | -12.8 (4.9) | -12.4 (3.2) | 0.882 | -14.5 (2.7) | -14.7 (5.5) | 0.896 | -13.5 (4.9) | -17.0 (4.4) | 0.591 |
| RVGLS (%) | -24.4 (-25.7, -22.1) | -23.8 (-26.1, -22.0) | 0.882 | -24.4 (3.6) | -25.1 (3.5) | 0.695 | -23.0 (4.2) | -25.3 (4.1) | 0.595 |
| RVGRS_SAX_ (%) | 22 (10) | 20 (7) | 0.882 | 24 (8) | 26 (13) | 0.821 | 23 (10) | 31 (14) | 0.595 |
| RVGRS_LAX_ (%) | 53 (16) | 53 (10) | 0.934 | 54 (16) | 60 (14) | 0.572 | 49 (16) | 54 (18) | 0.769 |
| RVGRS (%) | 38 (12) | 37 (7) | 0.882 | 39 (10) | 43 (11) | 0.572 | 36 (11) | 43 (16) | 0.651 |
| **Left atrium** |  |  |  |  |  |  |  |  |  |
| LALS (%) | 28.8 (18.7, 42.7) | 33.7 (24.6, 41.7) | 0.806 | 27.9 (7.5, 40.5) | 24.6 (21.5, 34.2) | 0.695 | 34.3 (15.4) | 45.8 (19.5) | 0.595 |
| **Right atrium** |  |  |  |  |  |  |  |  |  |
| RALS (%) | 38.4 (20.7) | 41.5 (16.9) | 0.882 | 35.4 (20.2) | 41.2 (13.0) | 0.572 | 31.1 (9.5) | 31.7 (9.2) | 0.932 |

Variables are expressed as numbers/total (percentages), mean (standard deviation), or median (interquartile range) for categorical, normally distributed, and non-normally distributed continuous variables. Abbreviations: GCS, global circumferential strain; GLS, global longitudinal strain; GRS, global radial strain; LA, left atrium; LAX, long-axis; LS, longitudinal strain; LV, left ventricle; n, number of subjects; N, total number of subjects; RA, right atrium; RV, right ventricle; SAX, short-axis; SV, stroke volume.
